# CD40/CD40L Signaling as a Promising Therapeutic Target for the Treatment of Renal Disease

**DOI:** 10.3390/jcm9113653

**Published:** 2020-11-13

**Authors:** Shungang Zhang, Joshua D. Breidenbach, Benjamin H. Russell, Jerrin George, Steven T. Haller

**Affiliations:** 1 Department of Medicine, University of Toledo College of Medicine and Life Sciences, Toledo, OH 43614, USA; Shungang.Zhang@rockets.utoledo.edu (S.Z.); Benjamin.Russell2@rockets.utoledo.edu (B.H.R.); Jerrin.George@rockets.utoledo.edu (J.G.); 2 Department of Medical Microbiology and Immunology, University of Toledo College of Medicine and Life Sciences, Toledo, OH 43614, USA; Joshua.Breidenbach@rockets.utoledo.edu

**Keywords:** CD40, chronic kidney disease, renal fibrosis

## Abstract

The cluster of differentiation 40 (CD40) is activated by the CD40 ligand (CD40L) in a variety of diverse cells types and regulates important processes associated with kidney disease. The CD40/CD40L signaling cascade has been comprehensively studied for its roles in immune functions, whereas the signaling axis involved in local kidney injury has only drawn attention in recent years. Clinical studies have revealed that circulating levels of soluble CD40L (sCD40L) are associated with renal function in the setting of kidney disease. Levels of the circulating CD40 receptor (sCD40), sCD40L, and local CD40 expression are tightly related to renal injury in different types of kidney disease. Additionally, various kidney cell types have been identified as non-professional antigen-presenting cells (APCs) that express CD40 on the cell membrane, which contributes to the interactions between immune cells and local kidney cells during the development of kidney injury. Although the potential for adverse CD40 signaling in kidney cells has been reported in several studies, a summary of those studies focusing on the role of CD40 signaling in the development of kidney disease is lacking. In this review, we describe the outcomes of recent studies and summarize the potential therapeutic methods for kidney disease which target CD40.

## 1. Introduction

Chronic kidney disease is the progressive loss of kidney function, which is prevalent in up to 13% of the global population and has become a major health burden characterized by reduced glomerular filtration rate and increased urinary albumin excretion [1,2]. As the loss of function advances, complete loss of kidney function progresses to kidney failure, or end-stage renal disease (ESRD), at which point dialysis and kidney transplant are the only treatment strategies. Each kidney consists of heterogeneous cell types and renal dysfunction is correlated with a complication of various physiological processes such as cardiovascular events, platelet activation, and inflammatory responses, among others [1,3,4,5].

**Figure 1:**
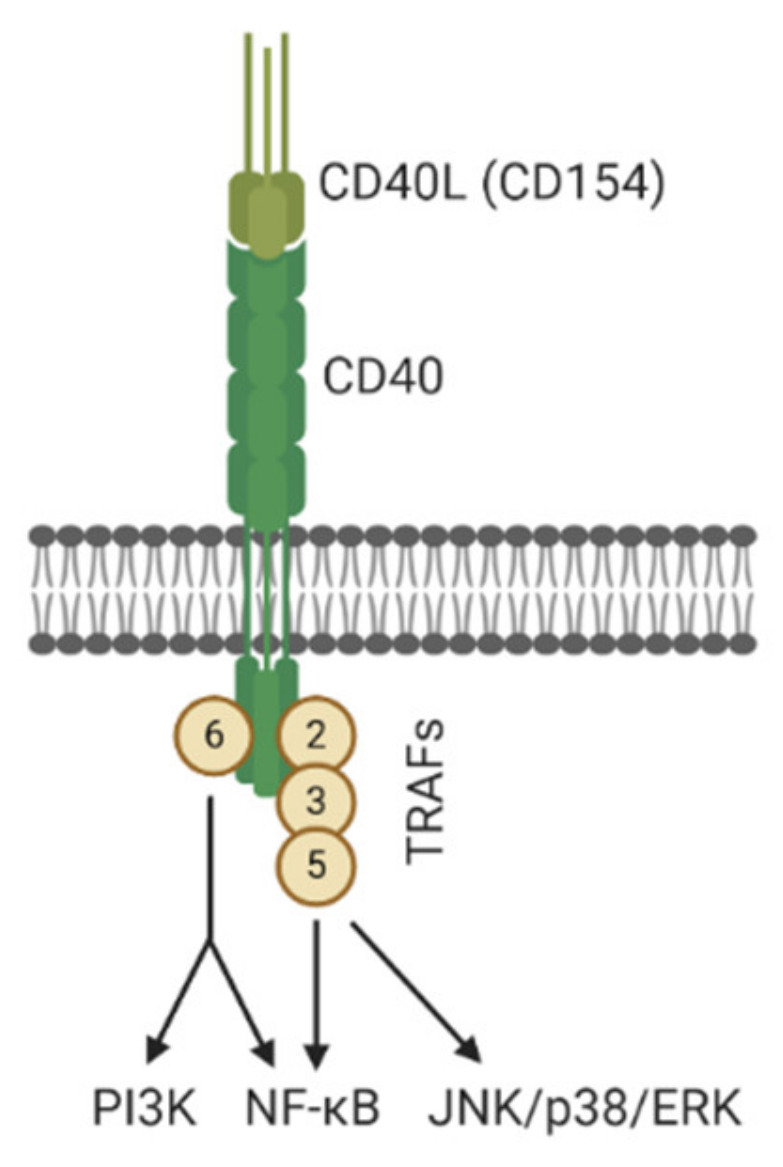
Illustration of CD40/CD40L regulated intracellular signaling. CD40 receptor activates different types of TRAFs upon stimulation and triggers signaling cascades including the PI3K, NF-κB, and p38/ERK pathways. (Figure created with BioRender.com).

The tumor necrosis factor receptor (TNFR) superfamily member 5, commonly referred to as the cluster of differentiation 40 (CD40), and its natural ligand, CD154 (CD40L), are well known as vital mediators of immunity and inflammation [6,7]. CD40 was initially found on antigen-presenting cells (APCs) required for the activation of B-cells during immune responses and is important in T-cell-dependent immunoglobulin class switching [8]. Activation of CD40 triggers a wide spectrum of signaling cascades through recruitment of TNFR-associated factors (TRAFs) and stimulating NF-kB signaling [9,10,11] (Figure 1). Moreover, numerous studies have demonstrated that CD40 is widely expressed in several different cell types, including B lymphocytes, macrophages, dendritic cells, endothelial cells, fibroblasts, and epithelial cells. Interactions between CD40 and CD40L regulate cell proliferation, differentiation, apoptosis, and inflammation [12,13]. The importance of CD40 in the development of renal disease and allograft rejection following kidney transplantation has focused on its role as an immune co-stimulatory molecule that is associated with adaptive immunity [14]. However, as our understanding of the involvement of inflammatory cells in the development of kidney disease has evolved, renal epithelial cells and podocytes have been demonstrated to express CD40 and are identified as antigen-presenting cells in which CD40 signaling renders important inflammatory responses on kidney pathology [15,16]. CD40 signaling in proximal tubule epithelial cells has been shown to enhance the production of pro-inflammatory and pro-fibrotic mediators and cause tubular injury [17,18]. In isolated podocytes, treatment with recombinant CD40L induced CD40 and matrix Metalloproteinase-9 expression, suggesting a contribution to glomerular basement membrane modifications [19]. As numerous reports are accumulating which implicate CD40/CD40L signaling in kidney disease, below we have summarized the recent studies to provide new insights and improve our understanding of the role of CD40 signaling in the development and progression of renal disease.

## 2. CD40 and CD40L as Markers of Renal Function in Kidney Disease

CD40 ligand (CD40L), or CD154, is primarily expressed by activated T-cells, activated B-cells, and platelets as a surface molecule of CD40 agonist [10]. However, CD40L also exists in a soluble form, soluble CD40 ligand (sCD40L), which is largely derived from activated platelets upon cleavage by matrix metalloproteinases and circulates in the plasma with intact biological activity [20,21]. Elevated levels of sCD40L have been identified in patients with stage 5 chronic kidney disease on hemodialysis [22]. Higher levels of circulating sCD40L have been shown to predict increased risk of cardiovascular (CV) morbidity and mortality in patients undergoing hemodialysis (HD) during a 24-month follow-up [3], whereas an additional study investigating HD patients demonstrated that circulating sCD40L levels have no effects on the CV events in a long-term follow-up (108 months) [23]. In addition, we have recently demonstrated that plasma levels of soluble CD40 receptor (sCD40), a circulating isoform of the CD40 receptor and an endogenous antagonist, predict progression of renal dysfunction in patients with chronic kidney disease (CKD) on multiple (≥3) anti-hypertensive medications and that sCD40L is significantly elevated in these settings [24] (Table 1).

As the primary source of sCD40L, hyperreactive platelets are associated with increased inflammatory activity in CKD patients [25,26]. Advanced oxidation protein products (AOPP), considered as renal pathogenic mediators [27,28], have been demonstrated to trigger human platelet CD40 ligand expression [29] and CD40 expression on dendritic cells [30]. Platelet-derived microparticles (PMPs) are the most abundant microparticles in circulation and increased PMPs are associated with diabetes mellitus, myocardial infarction, and CKD [31,32,33,34]. Mörtberg and colleagues have recently shown that circulating concentrations of PMPs expressing CD40 ligand were positively associated with the severity of chronic kidney disease, and the concentration of CD40 ligand + PMPs is inversely correlated with estimated glomerular filtration rate (eGFR) [35]. In other studies, CD40 ligand + PMPs of CKD patients can be reduced by the vitamin D2 derivative, paricalcitol [36], or lipid-lowering treatment (LLT) with simvastatin in the settings of diabetes mellitus (DM)-CKD [34].

Diabetic nephropathy, or diabetic kidney disease, is the most common cause of ESRD in diabetic patients [37,38,39]. Elevated levels of urinary sCD40L have been demonstrated in patients with type-1 diabetes with hyperglycemia [40]. Patients with diabetic nephropathy have higher plasma levels of sCD40L compared with normoalbuminuric patients, although sCD40L levels were not associated with a decline in kidney function [41]. In another study where kidney biopsies from patients with diabetic nephropathy were obtained, tubular expression of CD40 and infiltrating CD40L-expressing cells were significantly upregulated [42].

Associations between sCD40L and pathological parameters also have been reported in systemic lupus erythematosus (SLE) patients with chronic kidney disease in which sCD40L levels are elevated [43,44,45] and circulating levels of the CD40 receptor are negatively associated with eGFR [45]. Increased circulating sCD40L levels have also been described in patients with Shiga toxin-associated hemolytic uremic syndrome, where sCD40L levels are negatively correlated with levels of urea and creatinine [46]. Here, platelets were shown to be activated by Shiga toxin 2 (Stx2)-damaged human glomerular endothelial cells (HGEC), causing the release of sCD40L [46].

Serum levels of sCD40L are also higher in patients with nephrotic syndrome and focal segmental glomerulosclerosis (FSGS), although they are not correlated with proteinuria and eGFR [47]. Circulating sCD40L is thus identified to be a putative permeability factor in FSGS [48,49]. Interestingly, autoantibodies against CD40 have been identified in a study investigating recurrent FSGS after kidney transplantation. An analysis of circulating antibody panels revealed that antibodies against CD40 can help predict the risk of recurrent FSGS. Injection of the autoantibodies into mice was shown to induce proteinuria and injection of blocking antibody was found to alleviate the effect [50].

Renal artery stenosis is a major cause of hypertension and contributes to renal failure [51,52,53]. We have shown that patients with renal artery stenosis have high levels of circulating sCD40L compared to controls and that lower circulating levels of CD40 receptor (sCD40) were associated with a decline in renal function [4,54]. sCD40 has been proposed to act as an antagonist to the membrane CD40/CD40L interaction and is produced by proteolytic cleavage of the extracellular part of the CD40 molecule [55,56]. There is evidence indicating that accumulation of sCD40 is associated with altered humoral immune response in ESRD patients [57]. We recently investigated circulating levels of sCD40L and sCD40 in a CKD cohort and have shown that increased plasma sCD40L levels were associated with declined eGFR, and sCD40 levels were negatively associated with a reduction in eGFR. When the patients were stratified according to their plasma level of sCD40L (ligand) and sCD40 (receptor), subjects with high sCD40L/low sCD40 were found to predict a greater decline in eGFR at 1-year follow-up compared to subjects with low sCD40L/high sCD40, suggesting that sCD40 acts as a potential antagonist to CD40 signaling in this cohort [24].

Higher levels of circulating sCD40L have also been reported in ESRD patients [58]. However, conflicting results have been reported in an investigation of a separate smaller cohort of patients with ESRD [59]. Various risk factors contributing to ESRD have been identified and hemodialysis is a common treatment approach in this cohort [60,61]. Importantly, hemodialysis is associated with elevated markers of CD40 signaling. Patients with chronic renal failure after hemodialysis had increased CD40+ mononuclear cells in peripheral blood [62] and elevated sCD40L [63,64,65] and sCD40 [66,67] concentrations. The platelet inhibitor cilostazol was shown to reduce plasma sCD40L in CKD patients undergoing hemodialysis [68]. As prevalent evidence suggests high levels of sCD40L and activated CD40 signaling in renal disease patients, there has been a surge of reports on studies focusing on the processes regulated by CD40–CD40L interactions.

## 3. Implications of CD40 Expression in Immune Cells in Kidney Disease

CD40 expression in APCs such as B lymphocytes, macrophages, dendritic cells, endothelial cells, fibroblasts, and epithelial cells is well known and engagement of CD40–CD40L regulates a wide spectrum of cellular processes involving inflammatory responses [10,13]. Here, we summarize the studies focused on the CD40/CD40L axis in immune cells which contribute to local inflammation in kidney disease.

Activation of CD40 signaling was revealed to exacerbate autoimmunity and contribute to renal injury in the setting of systemic lupus erythematosus (SLE) [69]. Overexpression of CD40 on B-cells could be induced by estrogen followed by exacerbated lupus disease activity [70]. Additionally, defective signaling was identified in isolated B-cells from patients with SLE, and combination treatment of anti-IgM antibody and CD40L failed to reduce CXCR4 expression compared with healthy controls [71].

Peripheral Vδ2 T-cells in SLE patients also exhibited increased expression of CD40L [72]. CD40L on T-cells has been shown to contribute to the production of autoantibodies in SLE [73,74,75] and its overexpression was shown to be attributable to activated *CD40LG* gene due to X chromosome demethylation [76,77,78]. In a recent study, it was reported that impaired ERK-regulated DNA methyltransferase 1 (DNMT-1) and a transmethylation micronutrient-restricted (MR) diet caused DNA demethylation in the *CD40LG* gene regulatory regions of CD4+ T-cells. Importantly, this demethylation correlated with the development of SLE associated hematuria and glomerulonephritis in lupus-susceptible transgenic mice on an MR diet [79].

As the counterpart of the CD40 ligand, the *Cd40* gene was also found to be demethylated in inflammatory CD40+ monocytes in the setting of chronic kidney disease [80]. In fact, CD40+ monocytes have been proposed as a biomarker indicating severity of CKD [81]. Iron preparations and iron salt have been shown to reduce the surface expression of CD40 in monocytes from CKD patients [82]. Additionally, the CD40+ intermediate monocyte subsets were negatively correlated with eGFR and positively correlated with plasma/cellular homocysteine [80]. Homocysteine is a potential mediator of the S-adenosylmethionine/S-adenosylhomocysteine dyad and contributes to DNA hypomethylation [83], where it has been shown to promote CD40 expression in human endothelial cells [84]. In the setting of CKD, plasma homocysteine has been shown to be elevated, and both serum from CKD patients and homocysteine individually could promote differentiation of CD40+ intermediate monocytes in cultured human peripheral blood mononuclear cells (PBMCs). Inhibition of DNA methyltransferase-1 activity by homocysteine leads to hypomethylation of the CD40 promoter and the effect can be significantly attenuated by folic acid that counteracts homocysteine [80].

As a member of the TNF superfamily, CD40 activates both canonical and non-canonical NF-kB pathways [9]. NF-kB-inducing kinase (NIK) is a downstream mediator of the non-canonical NF-kB pathway and is implicated in the pathogenesis of SLE [85]. NIK deficiency in CD11c+ dendritic cells has been demonstrated to lead to impaired responses to CD40 activation [86]. The inhibitor of NF-kB signaling, NIK small molecule inhibitor (SMI), can inhibit CD40 signaling through non-canonical signaling depending on NIK in mouse and human B-cells. CD40L induced accumulation of p52 and expression of ICOSL (Inducible T-Cell Costimulatory Ligand) and IL-12p40 was suppressed after SMI treatment. In the mouse model of SLE, NIK inhibition suppressed the immune response and improved survival and renal function [85]. In addition to this, dendritic cell-like CD11c^+^ mononuclear phagocytes have been identified in glomeruli and the tubulointerstitium following TNF exposure, in which the surface expression of CD40 is upregulated [87].

In a recent study by our group, targeted disruption of *Cd40* in a model of salt-sensitive hypertension in rats (Dahl S) resulted in attenuated hypertensive renal dysfunction, characterized by decreased renal fibrosis, enhanced creatinine clearance, and reduced tubular injury, despite being just as hypertensive as the Dahl S wild-type [88]. In our model, in which CD40 function is abolished, we have shown significant reductions in collagen-1 in renal cortex tissue as well as significantly reduced proximal tubule expression of phospho-Lyn kinase and the pro-fibrotic mediator plasminogen activator inhibitor type-1 [88]. Recent work has shown that infiltrating T-cells contribute to the high salt-induced hypertensive phenotype of the Dahl S rat [89]. Our results are in agreement with this work as we demonstrate a significant increase in infiltrating CD3+ and CD8+ T-cells within the kidney in both *Cd40*-deficient and Dahl S rats following a high salt diet [88]. However, the *Cd40* deficiency resulted in significantly less renal fibrosis compared to Dahl S rats. Thus, while infiltrating T-cells may explain the persistent hypertension in the *Cd40* deficient rats, our data suggest that CD40 expressed within the kidney itself plays a central role in the development of renal fibrosis. Applying the Goldblatt two-kidney, one-clip (2K1C) model of ischemic renal disease in the Dahl S rats, in which a surgical clip (0.2 mm internal diameter) is placed around the left renal artery to induce ischemic renal injury, we reported significantly elevated levels of CD40 expression in the cortex of the ischemic kidney with extensive renal fibrosis [18]. Importantly, we noted significant improvements in renal function, including a decrease in urinary protein excretion and renal fibrosis in *Cd40*-deficient rats following the 2K1C procedure. Interestingly, reciprocal renal transplantation confirmed that local CD40 expressed in the kidney contributed to renal fibrosis in the ischemic kidney following the 2K1C procedure [18].

## 4. CD40 Signaling in Kidney Cells is Associated with Pathological Changes of Kidney Disease

Expression of CD40 has been identified in various kidney cell types, including podocytes [47], parietal epithelial cells [90], and proximal tubule epithelial cells [17]. Here, CD40 has been considered as an important mediator regulating inflammatory and fibrotic processes [15,17] and thus, more studies have focused on the role of CD40 in kidney cells and its implications involved in the development of kidney disease.

### 4.1. Renal Expression of CD40 Contributes to Inflammatory Responses in Kidney Disease

In a study using the doxorubicin (DOX)-induced nephropathy model in C57BL/6 mice, CD40 expression was shown to be elevated in renal glomeruli following injury [91]. Additionally, DOX nephropathy also promoted CD40-dependent inflammation by regulating Chemokine ligand 5 (CCL5) and Monocyte chemoattractant protein-1 (MCP-1). In the same study, when using the unilateral ureter obstruction model (UUO), CD40 activation increased expression of renal CD40, CCL5, and IL-12 [91]. In this study, bone marrow transplantation was performed to demonstrate that myeloid-derived CD40+ cells are the primary cell type contributing to renal inflammation after administration of CD40 activating mAb [91].

CD40 expression is also induced by IFN-γ in differentiated podocytes. Stimulation of podocytes with sCD40L leads to increased expression of MMP-9 [19]. In addition, IL-17 and CD40L can synergistically promote production of IL-6, MCP-1, CCL5, and NF-kB by podocytes [42]. An analysis of a circulating antibody panel in recurrent FSGS patients reveals that autoantibodies of CD40 derived from patients could disrupt the actin cytoskeleton of podocytes and cause podocyte depolarization [50]. Podocyte depolarization is a process associated with the suPAR (soluble urokinase plasminogen activator receptor)-β_3_ integrin signaling pathway and further examination demonstrated that CD40 autoantibodies and full length suPAR synergistically caused podocyte injury characterized by podocyte foot process effacement and process fusion/flattening in mice [92]. In another study using human primary podocytes, the authors demonstrated a mechanism in which sCD40L can transiently reduce the expression of nephrin, a transmembrane protein necessary for renal filtration barrier function, as well as inducing cytoskeleton reorganization which could contribute to altered glomerular permselectivity [47]. Evaluation of glomerular permselectivity in isolated rat glomeruli demonstrated that sCD40L treatment increased albumin permeability in glomeruli [47].

Tubulointerstitial fibrosis is an important process in the progression of kidney disease and renal tubular epithelial cells is one of the various cell types contributing to renal fibrosis [93]. In an in vitro study of human proximal tubular epithelial cells, CD40 ligand induced IL-6 expression and secretion under hypoxic conditions [94]. Transcriptional profiling by RNA-seq of isolated renal proximal tubule from the mouse model of UUO-induced renal fibrosis revealed that a section of proximal tubule acquires a proinflammatory phenotype during fibrosis, in which CD40 signaling was up-regulated [95].

In a murine model of SLE comparing CD40 antagonist antibody and the anti-inflammatory drug prednisolone, administration of CD40 antagonist antibody restored proteinuria, survival rate, and glomerular morphology, whereas prednisolone treatment alone was shown to have limited benefits [96]. Transcriptional profiling of the kidney tissue revealed that blocking CD40 also restored normal expression levels of nephrotic genes, which were not likely derived from infiltrated immune cells. Altered expression of nephrotic genes associated with glomerular or tubular cells was also identified [96]. These studies demonstrate the contributions of CD40 expression in parenchymal cells of the kidney to disease progression.

### 4.2. Renal Expression of CD40 is Tightly Regulated and Associated with Homeostatic Conditions

Renal tubular epithelial cells (RTECs) function as non-professional APCs, expressing many co-stimulatory molecules including CD40 [13,97]. Pathological conditions could activate the expression of co-stimulatory molecules and contribute to the interactions between RTECs and infiltrating immune cells [98,99]. As an example of this phenomenon, CD40 expression was increased in response to HBx gene expression in human proximal tubule epithelial cells (HK-2) [99] and in renal tubules in the mouse model of hepatitis B virus-associated glomerulonephritis (HBV-GN) [98]. This expression may be associated with HBx-induced Notch1 [100]. It has also been shown that HBx-stimulated HK2 cells can stimulate CD4^+^ T-cell proliferation and CD40L expression in co-culture experiments [98] (Table 1).

We have previously reported that cardiotonic steroids (CTS) promoted CD40 expression in the kidney cortex and proximal tubule epithelial cells. Cardiotonic steroids are ligands of the Na/K-ATPase and binding of CTS activates Na/K-ATPase/Src signaling through the Na/K-ATPase-α1 subunit [101]. CTS elevation in subjects with chronic kidney disease and its contribution to renal fibrosis have been reported by our group and others [102,103]. The cardiotonic steroid telocinobufagin (TCB) induced CD40 expression through the Na/K-ATPase/Src signaling pathway. Disruption of the interaction between the α1 subunit of Na/K-ATPase and Src kinase abrogated the effects of TCB and dysregulated basal CD40 signaling, and CD40 signaling was restored when the Na/K-ATPase/Src complex was rescued, suggesting that the Na/K-ATPase/Src complex regulates CD40 function in the proximal tubule epithelium [104].

Mesangial cells were also demonstrated to be non-professional APCs that activate CD4+ T-cells. CD40 expression in human mesangial cells can be induced by IFN-γ [105], soluble monosodium urate (MSU) [106], and low-density lipoprotein (LDL) [107] in vitro. Uric acid has been associated with CKD progression and it combines with sodium ions to form MSU [108,109]. Soluble MSU was reported to induce CD40 expression in a TLR4-dependent manner and contributes to human renal mesangial injury [106]. Similarly, lipid abnormalities are commonly associated with dysregulated systemic inflammation and oxidative stress in CKD patients, where reduced high-density lipoprotein (HDL) concentration and elevated low-density lipoprotein (LDL) has been reported [110,111]. LDL treatment of human mesangial cells was shown to stimulate CD40 expression by activating the IRE1α/IKK/NF-kB pathway. The ER stress inducer, tunicamycin, antagonizes LDL effects by suppressing the IRE1α/IKK/NF-kB pathway and attenuating CD40 expression [107].

**Figure 2:**
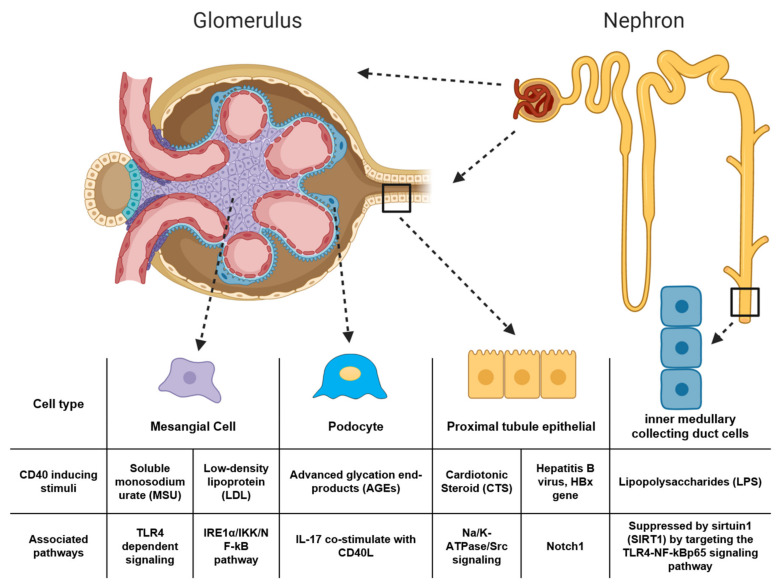
Summary of the known inducers of CD40 in mesangial cells [106,107], podocytes [42], proximal tubule epithelial cells [99,100,104], and the inner medullary collecting duct cells [112,113]. (Figure created with BioRender.com).

CD40 expression is also regulated in medullary cells and podocytes by different pathological stimuli. In primary culture of rat renal inner medullary collecting duct (IMCD) cells, LPS induced the expression of CD40 [112]. This effect was suppressed by sirtuin1 (SIRT1) by targeting the TLR4-NF-kBp65 signaling pathway [112] and miR-21 was found to be positively associated with SIRT1 expression and decreased CD40 expression in a separate study of TNF-alpha-induced IMCD cells [113]. In a study investigating diabetic nephropathy, advanced glycation end-products (AGEs) are used to mimic diabetic conditions and AGEs lead to up-regulation of TGF-β1, CD40, and IL-17 in cultured human podocytes where co-stimulation of IL-17 and CD40L strongly activates TGF-β1 and CD40 expression [42]. Studies of CD40 expression regulation in kidney cells are summarized in Figure 2.

## 5. Targeting CD40 Contributes to Therapeutic Treatments of Kidney Disease

Blocking of the CD40/CD40L pathways is well established in the study of facilitating allograft transplantation. Both antibodies targeting CD40 and CD40L have been reported for kidney transplantation [114,115]. CD154 antagonist antibody (MR1) has been used to prevent experimental renal ischemia reperfusion injury (IRI) [116]. Mice subjected to IRI under the condition of dual treatment of a MyD88 inhibitor and MR1 had completely restored survival rate, decreased serum creatinine (Cr), blood urea nitrogen (BUN), attenuated tubular damage and apoptosis, and reduced inflammatory cytokines in the kidney [116]. However, applications of anti-CD40L antibodies have limitations due to the development of thromboembolism [114,117]. In the setting of kidney disease, novel approaches such as DNA vaccination or siRNA against the CD40 gene may be a more promising approach.

DNA vaccination delivers plasmid DNA encoding the antigen and triggers an immune response [118,119]. DNA vaccination of CD40 targeting dendritic cells was reported to be protective of Heymann nephritis (HN) in an experimental rat model of autoimmune-mediated membranous nephritis [120]. Animals injected with the CD40 DNA vaccination developed anti-CD40 autoantibodies that could block B-cell activation and CD8+ T-cells proliferation [120]. Proteinuria, glomerulosclerosis, and tubular atrophy were all reduced following vaccination and histological analysis demonstrated reduced immune cell infiltration (CD4+ T-cells and CD68+ macrophages) as well as reduced IgG deposition in glomeruli [120]. The same group recently reported the use of CD40 DNA vaccination in experimental autoimmune glomerulonephritis. In this study, the CD40 DNA vaccine attenuated glomerulosclerosis and tubular atrophy by inhibiting Th17 differentiation and reducing immune cell infiltration (CD4+ T-cells, CD8+ T-cells, and CD68+ macrophages) [121].

RNA interference (RNAi) is an innate gene silencing mechanism at the post-transcriptional level [122]. The small inhibitory RNA (siRNA) technique has been developed based on the RNAi mechanism and has the advantage of specific gene knockdown. An siRNA approach against CD40 has been tested in the UUO model in mice, resulting in attenuated tubular dilation and interstitial fibrosis, reduced macrophages, and CD3+ T-cells infiltration [123]. Additionally, the study demonstrated a reduction in gene expression of MCP-1, iNOS, TGF-β1, and fibrosis matrix proteins such as fibronectin, MMP9, collagen IV, and α-SMA [123].

In a rat model of IRI, renal ischemia induced CD40 expression together with impaired renal function (elevated serum creatinine, acute tubular necrosis, tubular dilatation, interstitial edema and infiltrate) [124]. Injection of siRNA-CD40 attenuated renal injury and decreased CD68+ macrophages and CD3+ T-cells infiltration [124]. The anti-inflammatory cytokine IL-4 was upregulated and pro-inflammatory genes including CXCL9, CXCL10, CXCL11, and CCL2-5 were significantly decreased after siRNA treatment [124]. In the same study, siRNA-CD40 also attenuates previous overexpression of Socs3 and several genes related to the cell cycle [124]. Additional studies using the cold ischemia model of renal transplantation demonstrated that siRNA-CD40 administration reduced serum creatinine and renal injury, and significantly decreased levels of acute tubular necrosis [124].

Cholesterol conjugation to siRNA is known to improve cellular uptake. A cholesterol-conjugated anti-CD40-siRNA (Chol-siRNA) was reported to reduce the progression of lupus nephritis in a mouse model [125]. Chol-siRNA not only inhibited CD40 expression in interstitial, glomerular, and vascular compartments in the kidney, but attenuated pathological changes including proteinuria, extra-capillary proliferation, interstitial infiltrates, tubular atrophy, and interstitial fibrosis. Additionally, this treatment also resulted in reduced serum anti-dsDNA antibodies, serum cytokines IL12, TNF, IFNγ, MCP1, and IL6, as well as decreased C3 and IgG glomerular deposition [125].

A limitation associated with the use of siRNA is the lack of targeting ability and a systemic block of CD40 could impact the immune system, resulting in such effects as a reduction in the monocyte subset in the spleen and dysregulation of T-cells responses, as seen in the study discussed above [124]. Additionally, siRNA therapy was also shown to activate the immune response; as reported using an atherosclerosis (ATH) model of ApoE^-/-^ mice, anti-CD40 siRNA treatment only partially reversed the renal inflammation brought by scramble siRNA, indicating off-target side effects of siRNA [126]. Thus, the application of siRNA treatments needs to be further evaluated.

Antisense oligonucleotide (ASO) was reported to have a more favorable distribution into organs such as the kidney rather than lymphocytes. Generation 2.5 CD40 ASO treatment attenuated CD40-dependent inflammation in doxorubicin-induced nephropathy [91]. CD40 ASO administration improves glomerular nephropathy, interstitial and mesangial expansion, and granular tubular casts [91]. Renal injury markers such as CCL5, MCP-1, neutrophil gelatinase-associated lipocalin (NGAL), and connective tissue growth factor (CTGF) were also reduced after CD40 ASO treatment. In a UUO model, CD40 ASO treatment also attenuated inflammation by regulating CCL5 and IL-12p40 in a dose-dependent manner in both healthy and obstructed kidney [91]. However, with the preferable distribution of ASO to liver and kidney, the usage of ASO may have potential for nephrotoxic and hepatotoxic effects [127]. Various studies targeting CD40 for the treatment of kidney disease are summarized in Table 2.

## 6. CD40 and CD40L Blockade in Kidney Transplantation

The importance of CD40/CD40L signaling to kidney transplantation has been well studied in that CD40 acts as a co-stimulatory molecule associated with adaptive immunity as well as allograft rejection [14]. Usages of CD40 and CD40L blockade antibodies have been well reviewed in other reports [14,114,128,129]. CD40/CD40L contributes to antibody-mediated rejection (AMR) that is caused by donor-specific HLA antibody (DSA), although targeting CD40L alone is not sufficient to inhibit the production of DSA [114]. Usage of anti-CD40 mAb combined with other co-stimulation blockades has been reported as a promising therapy for prolonging graft survival [130]. Clinical studies have suggested the effectiveness of anti-CD40 monoclonal antibodies in kidney transplant patients to prevent rejection [115,131]. The anti-CD40 monoclonal antibody bleselumab was assessed in a phase 2 clinical study, in which the use of bleselumab combined with immediate-release tacrolimus (IR-TAC) demonstrated noninferiority to standard of care (SoC) condition in efficacy. However, assessment of drug safety shows that patient groups who are given bleselumab + MMF (mycophenolate mofetil) and bleselumab + IR-TAC have a higher rate of drug-related adverse events and hepatic events [115]. In another phase 1b study of kidney transplantation regarding pharmacokinetics and pharmacodynamics of bleselumab, no cytokine release syndrome or thromboembolic event was observed, while bleselumab was suggested to be more effective in combination with other immunosuppressants [131].

Another Fc-silenced, non-depleting anti-CD40 monoclonal antibody, CFZ533, has also been tested in nonhuman primate transplant studies [132,133], and recently, a phase 2a clinical trial has revealed that CFZ533 resulted in comparable efficacy as tacrolimus without thromboembolic events [134]. Patients following CFZ533 treatment demonstrated better renal function, less serious adverse events, and fewer infectious complications [134]. Additionally, ASKP1240 is another promising anti-CD40 monoclonal antibody that has been shown to be well tolerated without cytokine release syndrome or thromboembolic events in a phase I study in healthy subjects [135]. A separate clinical study in patients following kidney transplantation has demonstrated the safety, tolerability, pharmacokinetics, and pharmacodynamics of ASKP1240 [136].

As to the blockade of CD40L, a novel anti-human CD154 domain antibody lacking Fc binding activity possessing the advantage of no increased thromboembolism has been shown to prolong allograft survival in a study of nonhuman primate renal allograft rejection [137]. These studies suggest that blockade of the CD40/CD40L signaling may be an effective portion in the combination therapy for kidney transplantation.

## 7. Summary

The CD40/CD40L signaling axis plays important roles in the progression of a wide range of kidney diseases. Circulating levels of CD40L and the CD40 receptor provide promising insights on predicting the pathological and physiological conditions of kidney disease. Engagement of CD40 in immune cells is implicated in local renal inflammation and there has been increasing evidence demonstrating the importance of CD40 expression in kidney cells. CD40/CD40L signaling in various kidney cell types has demonstrable effects on the mediation of glomeruli permeability, interstitial inflammation, and fibrosis during kidney disease. Various disease-related factors may also regulate the expression of CD40 in kidney cells. Recently, CD40/CD40L has been elucidated as an important component in the interactions between immune cells and local kidney cells. Targeting CD40 has been demonstrated to be a promising approach and a potential therapeutic method for treating renal disease.

## Figures and Tables

**Table 1:** Summary of clinical studies demonstrating the associations between CD40/CD40L and kidney disease.

Disease Condition	Presence of CD40/CD40L	Associations with Kidney Function
Chronic kidney disease	CD40 ligand expression in platelet-derived microparticles (PMP)	Concentration of CD40 ligand + PMPs is positively associated with severity of CKD and inversely correlated with eGFR [35]
	Circulating sCD40L and sCD40	Increased plasma sCD40L levels are associated with declined eGFR, and sCD40 levels are negatively associated with the reduction in eGFR [24]
Diabetic nephropathy	Urinary sCD40L	Elevated in patients with type 1 diabetes [40]
	Circulating sCD40L	Circulating sCD40L is increased compared with normoalbuminuric patients but not associated with a decline in kidney function [41]
	Tubular expression of CD40	Upregulated in kidney biopsy [42]
	Infiltrating CD40L expressing cells	
Systemic lupus erythematosus (SLE)	Circulating sCD40L and sCD40	sCD40L levels are elevated [43,44,45] and circulating levels of the CD40 receptor are negatively associated with eGFR [45]
Shiga toxin-associated hemolytic uremic syndrome	Circulating sCD40L	Negatively correlated with levels of urea and creatinine [46]
Nephrotic syndrome and focal segmental glomerulosclerosis (FSGS)	Serum sCD40L	Increased in patients but not correlated with proteinuria and eGFR [47]
Renal artery stenosis	Circulating sCD40L and sCD40	Lower circulating levels of CD40 receptor (sCD40) are associated with a decline in renal function [4,54]

**Table 2:** Studies targeting CD40 in therapeutic treatments of kidney disease.

Approach	Distribution	Kidney Disease Model	Prevention Effects	Drawbacks
CD40L antagonist (MR1)	Non-specific	Renal ischemia reperfusion (IRI) in mouse	MR1 in combination with MyD88 inhibitor restored survival rate, decreased serum creatinine (Cr), blood urea nitrogen (BUN), attenuated tubular damage and apoptosis, and reduced inflammatory cytokines in the kidney [116]	Risk of thromboembolism events by CD40L antibody
DNA vaccination	Dendritic cells	Heymann nephritis (HN) in rats	Block B-cell activation and CD8+ T-cells proliferation, reduced proteinuria, glomerulosclerosis, tubular atrophy, immune cell infiltration and IgG deposition [120]	Targeting dendritic cells, limited to autoimmune-mediated kidney disease
		Autoimmune glomerulonephritis in rats	Attenuated glomerulosclerosis and tubular atrophy; reduced immune cell infiltration [121]	
siRNA	Non-specific	Unilateral ureteral obstruction (UUO) in mouse	Attenuated tubular dilation and interstitial fibrosis, reduced macrophage and CD3+ T-cells infiltration, and reduced gene expression of pro-fibrotic cytokines [123]	Lack of ability to target distribution, triggers immune responses and has off-target side effects
		Renal ischemia reperfusion (IRI) in rats	Attenuated renal injury, CD68+ macrophages and CD3+ T-cells infiltration, pro-inflammatory genes expression, and suppressed overexpression of genes related to cell cycle [124]	
		Lupus nephritis in mouse	Cholesterol-conjugated anti-CD40-siRNA attenuated proteinuria, extra-capillary proliferation, interstitial infiltrates, tubular atrophy, and interstitial fibrosis; reduced serum anti-dsDNA antibodies and circulating pro-inflammatory cytokines [125]	
Generation 2.5 antisense oligonucleotide (ASO)	Favorable distribution into organs including kidney	Doxorubicin (DOX)-induced nephropathy and UUO model in mouse	Improved glomerular nephropathy, interstitial and mesangial expansion, granular tubular casts, and reduced renal injury markers [91]	Potential for nephrotoxicity and hepatotoxicity
